# Epigenetic silencing of lncRNA *MORT* in 16 TCGA cancer types

**DOI:** 10.12688/f1000research.13944.1

**Published:** 2018-02-21

**Authors:** Lukas Vrba, Bernard W. Futscher

**Affiliations:** 1 The University of Arizona Cancer Center, Tucson, AZ, 85724, USA; 2 Department of Pharmacology & Toxicology, College of Pharmacy, The University of Arizona, Tucson, AZ, 85724, USA

**Keywords:** DNA Methylation, Gene Silencing, lncRNA, ncRNA, lincRNA, MORT, ZNF667-AS1, Epigenetics

## Abstract

We have previously described a hominid-specific long non-coding RNA, *MORT* (also known as *ZNF667-AS1*, Gene ID: 100128252), which is expressed in all normal cell types, but epigenetically silenced during cancer-associated immortalization of human mammary epithelial cells.  Initial analysis of The Cancer Genome Atlas (TCGA) showed that 15 of 17 cancer types, which represent the 10 most common cancers in women and men, display DNA methylation associated *MORT *silencing in a large fraction of their tumors.  In this study we analyzed *MORT* expression and DNA methylation state in the remaining 16 TCGA cancer types not previously reported.  Seven of the 16 cancer types showed DNA methylation linked *MORT *silencing in a large fraction of their tumors.  These are carcinomas (cervical cancer, and cancers of esophagus, stomach, and bile duct), and the non-epithelial tumors mesothelioma, sarcoma, and uterine carcinosarcoma.  Together with the findings from our previous report, *MORT* expression is silenced by aberrant DNA methylation in 22 of 33 of TCGA cancer types.  These 22 cancers include most carcinoma types, blood derived cancers and sarcomas.  In conclusion, results suggest that the *MORT* gene is one of the most common epigenetic aberrations seen in human cancer.  Coupled with the timing of *MORT* gene silencing during *in vitro* epithelial cell immortalization and its occurrence early in the temporal arc of human carcinogenesis, this provides strong circumstantial evidence for a tumor suppressor role for *MORT*.

## Introduction

*MORT* was originally found as a transcript silenced during *in vitro* immortalization of human mammary epithelial cells ^1^. Like a significant majority of lncRNAs, *MORT*’s molecular function remains enigmatic. The *MORT* gene is specific to higher primates, is expressed in all normal human cell types, and *MORT* RNA is located predominantly in the cytoplasm ^1^. Analysis of *MORT* expression and the DNA methylation state of its promoter in 17 cancer types from The Cancer Genome Atlas (TCGA) ^2^, which represent the 10 most frequent cancers in males and females, showed *MORT* is epigenetically silenced in 15 of 17 these cancers ^1^. Based on the data from the original *in vitro* study ^1^, we predicted epigenetic *MORT* silencing occurs early in human carcinogenesis and therefore could be seen in premalignant lesions, such as ductal carcinoma *in situ* of the breast and colonic adenomas. We used data from clinical samples from published genomic data sets ^3– 8^ to address this possibility, and indeed, *MORT* loss occurs prior to or at the stage of pre-malignancy and not thereafter ^9^. Taken together these facts suggest that *MORT* transcript has a tumor suppressive role and is not simply an epigenetic “passenger error.”

Since our previous analysis of *MORT* in TCGA datasets was not exhaustive and only reported on 17 out of 33 TCGA cancer types, the goal of this short study was to extend our earlier work and complete the analysis of *MORT* DNA methylation associated gene silencing in the final 16 TCGA cancer types.

## Methods

We integrated the *MORT* expression level and the DNA methylation state of its promoter region using TCGA data as described before ^1^. The Illumina HiSeq RNA-seq and HumanMethylation450 DNA methylation data for samples of 16 TCGA cancer types listed in Table 1 were downloaded from the GDC data portal. The data were analyzed in the R programming environment, version 3.4.2 ^10^. The mean RNA-Seq rpkm values for the two exons constituting the *MORT* RNA were plotted against the mean DNA methylation beta value of the 7 CpGs from the *MORT* promoter region for the individual samples of each cancer type. The Spearman correlation coefficient rho between the *MORT* RNA level and the DNA methylation of *MORT* promoter was calculated using the function cor.test.

**Table 1.** The 16 TCGA cancer types analyzed in this study. The numbers of primary tumor and normal samples for which both the *MORT* RNA expression and the *MORT* promoter DNA methylation data were available are listed. *DNA methylation data from HumanMethylation27 platform that covers 2 CpGs out of 7 CpGs covered by HumanMethylation450 were used.

TCGA Cancer Type Name	Abbreviation	Tumor samples	Normal samples
adrenocortical carcinoma	ACC	79	0
cervical squamous cell carcinoma and endocervical adenocarcinoma	CESC	304	3
cholangiocarcinoma	CHOL	36	9
esophageal carcinoma	ESCA	184	9
glioblastoma multiforme	GBM	51	1
kidney chromophobe	KICH	66	0
brain lower grade glioma	LGG	516	0
mesothelioma	MESO	87	0
ovarian serous cystadenocarcinoma	OV *	295	0
pheochromocytoma and paraganglioma	PCPG	179	3
sarcoma	SARC	259	0
stomach adenocarcinoma	STAD	373	0
testicular germ cell tumors	TGCT	150	0
thymoma	THYM	120	2
uterine carcinosarcoma	UCS	57	0
uveal melanoma	UVM	80	0

## Results and discussion

**Figure 1.**
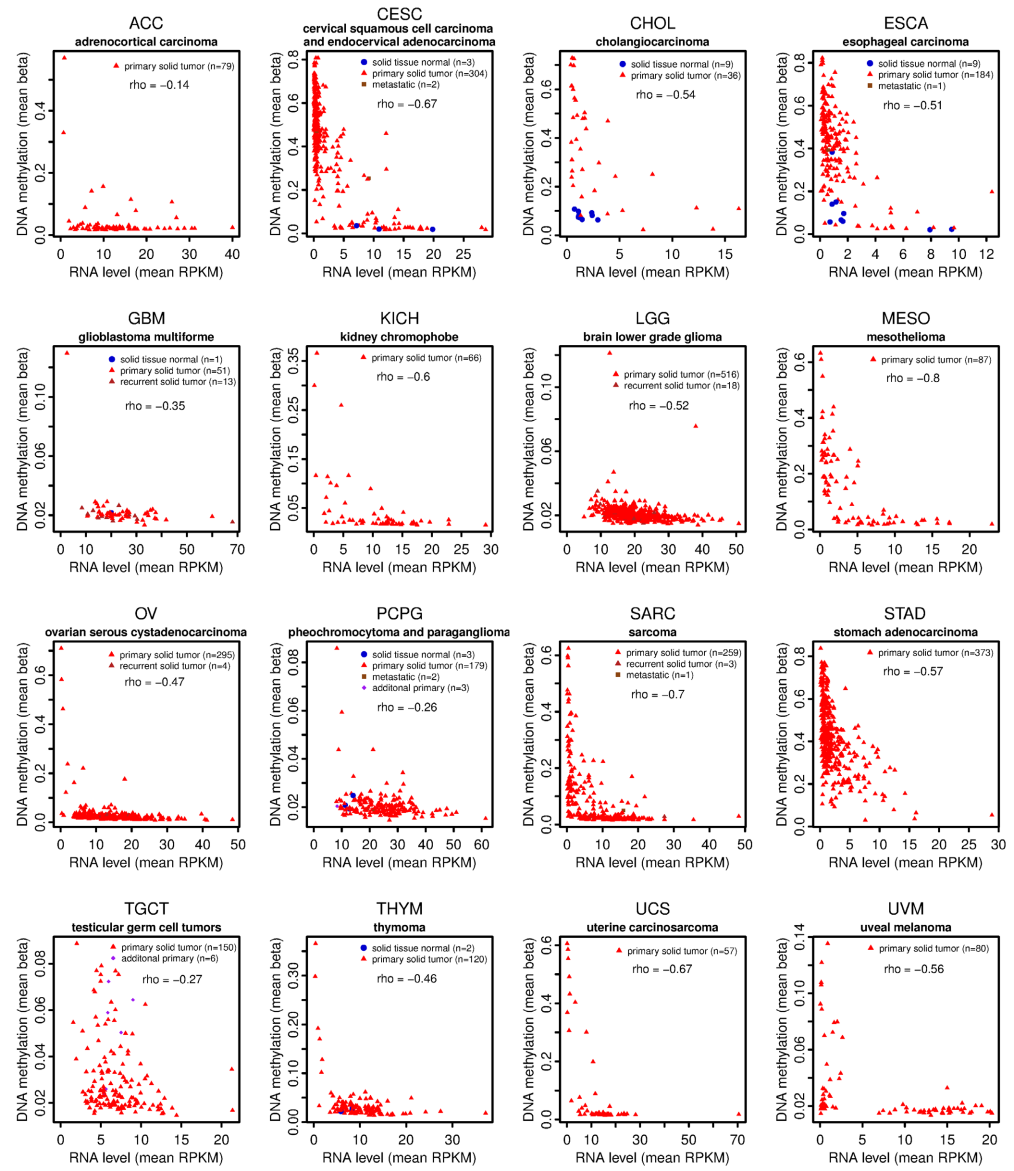
Integration of the *MORT* expression and the *MORT* promoter DNA methylation TCGA data for 16 tumor types. The x-axis shows the *MORT* expression level according to RNA-seq and y-axis shows the level of *MORT* promoter DNA methylation according to Illumina HumanMethylation450 microarray. The correlation coefficient rho between the *MORT* expression and the DNA methylation of *MORT* promoter for each tumor type is displayed. The OV has a very low number (10) of samples analyzed by the HumanMethylation450 platform, therefore the data from the HumanMethylation27 platform that covers 2 CpGs out of 7 CpGs covered by HumanMethylation450 were used.

Seven of sixteen analyzed cancer types (CESC, CHOL, ESCA, MESO, SARC, STAD, and UCS) show strong *MORT* silencing by DNA methylation ( Figure 1). The negative correlation rho between *MORT* expression and DNA methylation in these cancers is below -0.5; the DNA methylation level in some tumor samples of these cancers exceeds 0.5 beta (> 50% DNA methylation), and a large fraction of the tumor samples in these cancer types have very low to no *MORT* expression level ( Figure 1). The correlation of *MORT* expression and promoter DNA methylation in the remaining nine cancer types is also negative; however, the maximum level of the DNA methylation of *MORT* promoter in some of these cancers is either very low (UVM), or a very few tumor samples have *MORT* silenced (ACC, KICH, OV, and THYM), and some of the cancer types (GBM, LGG, PCPG, and TGCT) do not appear to display *MORT* gene silencing ( Figure 1).

The analysis presented shows DNA methylation associated *MORT* gene silencing in 7 of 16 TCGA cancer types. Compared to the 17 TCGA cancer types presented in our original study, ^1^ most the 16 cancer types presented here lack their respective normal tissues samples and some of them have lower amounts of tumor samples ( Table 1). Nevertheless, the distribution of *MORT* expression and DNA methylation data in tumor samples clearly indicates *MORT* silencing in multiple cancer types ( Figure 1).

Cervical tumors (CESC) have high proportion of *MORT* silencing ( Figure 1); more than 75% of 304 cervical tumor samples have *MORT* promoter DNA hypermethylated and *MORT* silenced. Using TCGA data, a recent study found *MORT* downregulated in cervical cancer ^11^, but surprisingly did not report on or hypothesize potential mechanisms for this transcriptional repression. Here we confirm and extend their initial analysis of *MORT* silencing in cervical cancer and show further that this silencing is strongly linked to aberrant DNA methylation of the *MORT* promoter.

Combined together with the findings from our previous report ^1^, Table 2 shows *MORT* is silenced by DNA methylation in a super majority of TCGA cancer types (22 of 33). *MORT* loss occurs predominantly due to epigenetic silencing and increased DNA methylation of its promoter in breast cancer ^9^. This could likely be extended to all 22 cancer types with the high fraction of *MORT* negative samples and the high correlation between *MORT* RNA level and *MORT* promoter DNA methylation, where *MORT* likely plays a tumor suppressive role. The other 11 cancer types, with a little to no *MORT* silencing, might have tumor suppressive pathway, where *MORT* is involved, interrupted elsewhere and/or *MORT* may play some additional vital role in tissues these tumors originate from - e.g. prostate, thyroid, brain, testes, or ovary - since these tissues typically have the highest levels of *MORT* RNA ^1^.

**Table 2.** Summary of *MORT* silencing in all 33 TCGA cancer types. The cancer types with *MORT* silencing in a large fraction of tumor samples are indicated. Results from this study are indicated (*), results from our previous report (ref 1) are indicated (**).

Abbreviation	TCGA cancer type name	*MORT* silencing
ACC	adrenocortical carcinoma	No *
BLCA	bladder urothelial carcinoma	Yes **
BRCA	breast invasive carcinoma	Yes **
CESC	cervical squamous cell carcinoma and endocervical adenocarcinoma	Yes *
CHOL	cholangiocarcinoma	Yes *
COAD	colon adenocarcinoma	Yes **
DLBC	lymphoid neoplasm diffuse large b-cell lymphoma	Yes **
ESCA	esophageal carcinoma	Yes *
GBM	glioblastoma multiforme	No *
HNSC	head and neck squamous cell carcinoma	Yes **
KICH	kidney chromophobe	No *
KIRC	kidney renal clear cell carcinoma	Yes **
KIRP	kidney renal papillary cell carcinoma	Yes **
LAML	acute myeloid leukemia	Yes **
LGG	brain lower grade glioma	No *
LIHC	liver hepatocellular carcinoma	Yes **
LUAD	lung adenocarcinoma	Yes **
LUSC	lung squamous cell carcinoma	Yes **
MESO	mesothelioma	Yes *
OV	ovarian serous cystadenocarcinoma	No *
PAAD	pancreatic adenocarcinoma	Yes **
PCPG	pheochromocytoma and paraganglioma	No *
PRAD	prostate adenocarcinoma	No **
READ	rectum adenocarcinoma	Yes **
SARC	sarcoma	Yes *
SKCM	skin cutaneous melanoma	Yes **
STAD	stomach adenocarcinoma	Yes *
TGCT	testicular germ cell tumors	No *
THCA	thyroid carcinoma	No **
THYM	thymoma	No *
UCEC	uterine corpus endometrial carcinoma	Yes **
UCS	uterine carcinosarcoma	Yes *
UVM	uveal melanoma	No *

In summary, our findings show that the *MORT* gene is one of the most common epigenetic aberrations seen in human cancer. Coupled together with *MORT* silencing occurring early in the temporal arc of human carcinogenesis it strongly supports a tumor suppressive role for *MORT*.

## Data availability

Illumina HiSeq RNA-seq and HumanMethylation450 DNA methylation data for TCGA cancer types used in the present study can be downloaded from the GDC data portal.
