# Supplementary material for: Epigenetic silencing of lncRNA MORT in 16 TCGA cancer types
Source: F1000Res. 2018 Feb 21;7:211. [Version 1] doi: 10.12688/f1000research.13944.1 (PMC5883388; doi:10.12688/f1000research.13944.1)
Supplement: Peer review history

# Peer review: Referee response for version 1

**DOI:** 10.5256/f1000research.15158.r31592

**Published:** 2018-04-03

**Authors:** Alexander Dobrovic

**Affiliations:** 1 Translational Genomics and Epigenomics Laboratory, Olivia Newton-John Cancer Research Institute, Heidelberg, Victoria, Australia

This brief article provides an analysis of MORT epigenetic silencing across 16 TCGA tumour types not previously reported by the authors enabling assessment across all TCGA tumour types. MORT silencing seems to  be a key early event in carcinogenesis. This indicates that further studies of MORT biology are required as well as investigation of MORT methylation as a potential biomarker.

I have read this submission. I believe that I have an appropriate level of expertise to confirm that it is of an acceptable scientific standard.

# Peer review: Referee response for version 1

**DOI:** 10.5256/f1000research.15158.r31980

**Published:** 2018-04-03

**Authors:** Gwen Lomberk

**Affiliations:** 1 Division of Research, Department of Surgery, Medical College of Wisconsin, Milwaukee, WI, USA

STRENGTHS: The authors utilize the TCGA database to extend their previous studies on MORT that has been primarily characterized in breast cancer, but also observed to undergo silencing in 15 out of the 17 most common cancers. The current work takes this analysis deeper into the 33 TCGA cancer types and perform a more thorough analysis of the DNA methylation associated with the 16 cancer types evaluated here.

 SUGGESTIONS FOR IMPROVEMENT:

  -The authors make a bold statement that “the MORT gene is one of the most common epigenetic aberrations seen in human cancer”. This statement is not supported by the data presented. In order to make a statement of this level, the authors would have to provide comparisons to other documented genes that undergo a high frequency of epigenetic alteration. 

 -This statement becomes more difficult to make when considering that several of the tumors present do not have normal counterparts for comparison. We therefore do not know if those tissue types whether the DNA methylation is abnormal or typical for the tissue type. 

 -Authors should consider revising some of the scales on the graphs in Figure 1 to highlight the lack of methylation (for example, in testicular germ cell tumors). Similarly, the cutoff for methylation could be shown by a dotted line or similar feature. 

 -The location of methylation relative to the MORT gene would be informative. Of the tumor types that are methylated, are the same regions methylated across types? 

 -The authors should be cautious not to overstate their conclusions throughout the text since the conclusions are speculative without experiments to support their statements. Nevertheless, these correlations present interesting speculation and avenues for further investigations.

I have read this submission. I believe that I have an appropriate level of expertise to confirm that it is of an acceptable scientific standard.

# Peer review: Referee response for version 1

**DOI:** 10.5256/f1000research.15158.r31096

**Published:** 2018-03-01

**Authors:** Adam Karpf

**Affiliations:** 1 Eppley Institute and Fred & Pamela Buffett Cancer Center, University of Nebraska Medical Center, Omaha, NE, USA

The authors present a straightforward study of DNA methylation and RNA expression of the lncRNA MORT in 16 TCGA cancer types not previously reported.  Most of the paper is clearly presented and the conclusions are reasonable.  Suggestions for improvement are presented below.

 1) Statements are made regarding MORT being “one of the most common” epigenetic aberrations seen in human cancer.  No support for this statement is presented.

 2) The classification of some tumor types as showing “strong” MORT silencing by DNA methylation, and other tumor types as not showing this, is arbitrary.  A quantitative definition is needed.

 3) Fig 1 might be clearer if presented in two panels, subdivided by whether the quantitative definition of “strong silencing” is met.  The same is true for Table 2, which could be subdivided into two parts.

 4) Since normal samples were not available for many of the tumors profiled, a more accurate statement might be “DNA methylation regulation” rather than “DNA methylation silencing,” as reduced expression in tumors as compared to normal tissues was not shown.

 5) A diagram showing the MORT gene and the position of the methylation sites examined by the TCGA would be helpful.

 6) Can the authors reference any data, e.g. from other publications, showing that DNA methylation actively suppresses MORT expression, by for example turning on MORT expression using treatment with a DNA methyltransferase inhibitor?

 7) In the introduction, the statement “prior to or at the stage of pre-malignancy and not thereafter” is confusing, and gives the impression that MORT becomes hypomethylated, or its expression is elevated, in tumors as compared to pre-malignant lesions.  A better way to phrase this would be to say e.g. that “MORT is repressed in pre-malignant lesions and remains repressed in tumors.”

 8) The final sentence in the discussion uses the wording “strongly supports” when “suggests” would be more accurate, given the absence of functional data showing tumor suppression by MORT.

I have read this submission. I believe that I have an appropriate level of expertise to confirm that it is of an acceptable scientific standard.
